# JPEG Image Enhancement with Pre-Processing of Color Reduction and Smoothing

**DOI:** 10.3390/s23218861

**Published:** 2023-10-31

**Authors:** Akane Shoda, Tomo Miyazaki, Shinichiro Omachi

**Affiliations:** Graduate School of Engineering, Tohoku University, Sendai 9808579, Japanmachi@ecei.tohoku.ac.jp (S.O.)

**Keywords:** pre-processing, image compression, image enhancement, deep learning, JPEG, signal processing

## Abstract

JPEG is the international standard for still image encoding and is the most widely used compression algorithm because of its simple encoding process and low computational complexity. Recently, many methods have been developed to improve the quality of JPEG images by using deep learning. However, these methods require the use of high-performance devices since they need to perform neural network computation for decoding images. In this paper, we propose a method to generate high-quality images using deep learning without changing the decoding algorithm. The key idea is to reduce and smooth colors and gradient regions in the original images before JPEG compression. The reduction and smoothing can suppress red block noise and pseudo-contour in the compressed images. Furthermore, high-performance devices are unnecessary for decoding. The proposed method consists of two components: a color transformation network using deep learning and a pseudo-contour suppression model using signal processing. The experimental results showed that the proposed method outperforms standard JPEG in quality measurements correlated with human perception.

## 1. Introduction

Image compression is crucial for high-speed data transfer and reduces memory usage. The international standard for still image coding, JPEG [1], is still widely used because of its simple coding process and low computational complexity. However, JPEG images often deteriorate. Thus, JPEG image enhancement is an essential technique.

Many deep-learning-based methods have been developed in the field of image processing. They enhanced image quality significantly. For example, the methods in references [2,3,4,5,6,7] enhanced JPEG-coded images using neural networks. However, they required substantial computational resources to generate high-quality images since a large neural network model needs to be performed. Thus, only limited devices can use such image enhancement. In other words, smartphones are unavailable.

Image compression with pre-processing has been studied to avoid post-processing for artifact reduction in compressed images. The methods aim to reduce compression artifacts through pre-processing [8,9,10,11,12]. Broadly, the pre-processing methods can be divided into two approaches: hand-crafted and learned pre-processing, respectively. Human experts use a non-linear diffusion filtering [8] and pre-processing optimized to the display device [9]. The learned pre-processing methods are developed using a neural network [10,11,12]. Both pre-processing approaches successfully improve the visual quality of compression images.

The motivation of this work is to enhance the visual quality of JPEG compressed images using pre-processing. The advantage of pre-processing is that a huge computation is required only for the image encoder. Therefore, lightweight devices can obtain high-quality JPEG images in the same way as they would a standard JPEG image. We aim to mitigate degradation, such as red block noise and pseudo-contour. These degradations are the main reason for deteriorating visual quality. Our observation and analysis (the details are described in Section 3) show that low saturation and low-frequency regions cause the degradation. Thus, we use a color transformation and a smoothing model to alleviate the degradation.

**Figure 1:**
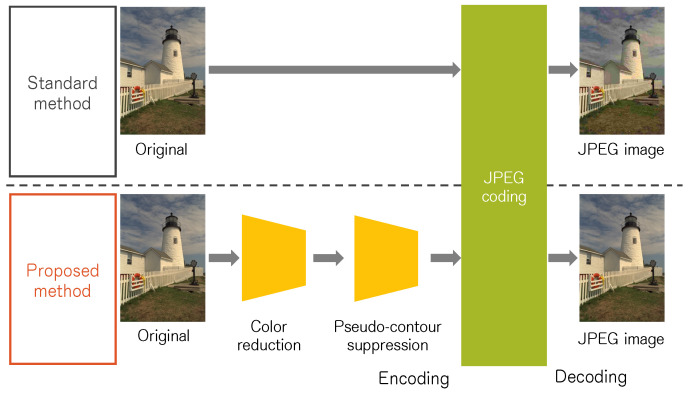
Strategies of standard JPEG coding and the proposed method.

The contribution of this work is the pre-processing of color reduction and smoothing for JPEG image enhancement. We propose a pre-processing method composed of the learned and hand-crafted approaches. Specifically, a learned neural network model reduces colors to alleviate red block noise. Furthermore, an artifact of pseudo-contour is suppressed using a smoothing algorithm. Figure 1 shows the strategies of standard JPEG coding and the proposed method. As with pre-processing methods [8,9,10,11,12], any modifications are not required by a compression algorithm such as JPEG. Only the encoder devices need pre-processing computation. Therefore, lightweight devices can obtain high-quality JPEG images in the same way as the standard JPEG image. The proposed method is a substitute for a JPEG encoder. Thus, an example application is an image compression module used in an image streaming server, such Unsplash, Instagram, etc.

We conducted numerical and subjective experiments to verify the effectiveness of the proposed method. The results showed that the proposed method improved JPEG image quality.

## 2. Related Work

Image enhancement aims to produce better-quality images for human vision. Specifically, there are applications such as JPEG artifact removal, denoising, and super-resolution. In these tasks, many learning-based methods have been developed in recent years.

For JPEG artifact removal, Dong et al. [2] developed the first deep learning method based on a super-resolution network, SRCNN [13]. Zhang et al. [14] used batch normalization [15] and residual learning [16] to facilitate the training process and improve the performance of image enhancement tasks. Fu et al. developed a deep convolutional sparse coding network that combines model-based methods and deep learning [3]. Other methods [4,5,6,7] trained convolutional networks to exploit redundancy in both pixel and DCT domains. Ehrlich et al. utilized quantization tables as prior information to train the network [17]. Also, a flexible blind convolutional neural network [18] used quality factor attention blocks to correct artifacts. However, these methods require a decoder with sufficient computational resources, which are not daily used devices.

There is also the task of soft color segmentation, which decomposes an image into multiple RGBA layers containing only specific colors. Various methods have been developed [19,20,21]. Akimoto et al. optimized decomposed layers for image reconstruction [20]. The method consists of three parts: palette selection, α-layer estimation, and residual color estimation. The palette selection extracts representative colors from the image and generates a color palette for each color. The module of α-layer estimation calculates α values of the generated color palette. After processing the α-layer, the residual color estimation estimates the residual colors between the colors of the original and the color palette. For each pixel in the final RGBA layer, the RGB value is the sum of the color palette and its color residuals at that pixel. The α value is obtained from the generated α-layer. The decomposed RGBA layers can be used for image editing, such as recoloring or compositing.

Inspired by the method of reconstructing images from specific colors [20], we produce a JPEG compressed image from an input image, the colors of which are reduced through pre-processing. Furthermore, color reduction can suppress red block noise in the compressed images. Thus, we can enhance the visual quality of the JPEG images. We stress that the decoding does not involve any neural network computation and can be handled in the same way as ordinary encoded images.

## 3. Motivation and Analysis of JPEG Images

**Figure 2:**
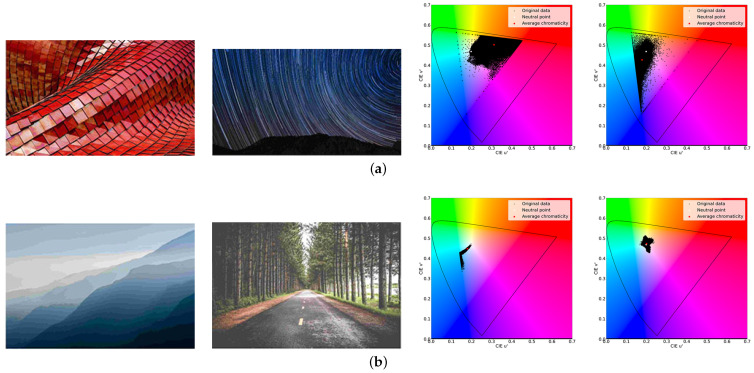
Images manually divided into good and poor visual groups and their color distributions in u${}^{\prime }$v${}^{\prime }$ uniform chromaticity diagrams. (**a**) Good visual group; (**b**) Poor visual group.

We analyze JPEG images to reveal the main causes of degradation. Specifically, we used images of the publicly available dataset, CLIC2021 (http://clic.compression.cc/2021/tasks/index.html, accessed on 1 February 2023). The images are high-quality images of natural scenes, buildings, animals, etc. Thus, we applied JPEG compression to the images with a quality value of 10. Then, we manually divided the images into good and poor visual groups and investigated their characteristics. Figure 2 shows images of good and poor groups with their color distributions in u’v’ uniform chromaticity diagrams. The images in the good group tend to have clear colors, high saturation, and abundant high-frequency components. On the other hand, the poor group contains low color saturation and few high-frequency components. There are degradations, such as unnatural tints caused by red block noise appearing in low-saturation images. Furthermore, pseudo-contours occur in the images with a few high-frequency components.

### 3.1. Discussion of Red Block Noise

In JPEG-coded images, block noise is generated by dividing an image into 8×8 pixels and applying a discrete cosine transform. Since human vision is sensitive to the red component, block noise, especially in the red component, has a significant impact on the visual quality of the image.

As shown in Figure 2, we illustrate color distribution in $u^{\prime }v^{\prime }$ uniform chromaticity diagrams to visualize the color distribution. The center of the diagram, $\left(u^{\prime },v^{\prime }\right)=\left(0.20,0.47\right)$, is called the neutral point, which represents the achromatic color (pure white). The saturation increases if a color moves outward from the neutral point. The distribution of the good visual group is located in the outer part of the diagram, indicating that the images contain many highly saturated colors. In contrast, the poor group squeezes colors at the neutral points. Thus, the images are composed mainly of colors with low saturation.

**Figure 3:**
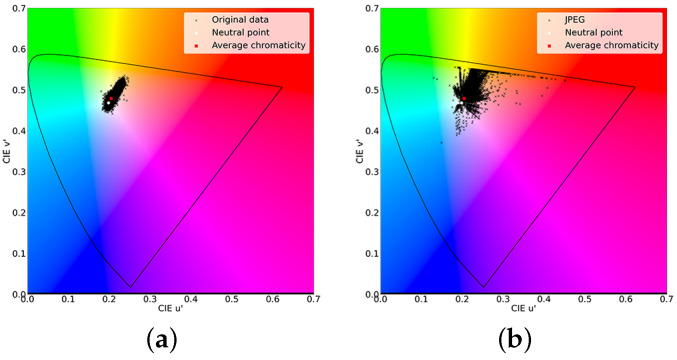
Color distributions before and after JPEG coding. (**a**) Before; (**b**) After.

Figure 3 shows the color distributions of an uncompressed image and its JPEG image, in which red block noise occurs. The color distribution moved outward after JPEG compression. More precisely, the colors were densely squeezed at the achromatic colors before JPEG coding and then dispersed to the outside after JPEG coding. Considering the color change after JPEG coding in the image domain, colors were the same for human perceptions before compression. However, the colors become independent after compression, resulting in color block noise. Furthermore, a color changes along the $u^{\prime }$ axis and becomes red block noise in the image domain.

**Figure 4:**
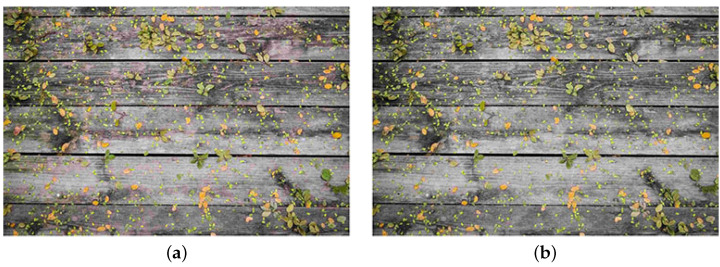
Effect of color reduction for red block noise in JPEG images. (**a**) Without color reduction; (**b**) With color reduction.

A solution for suppressing red block noise is color reduction before JPEG compression. Color reduction replaces colors with representative colors. Thus, color distribution suppresses the effect of JPEG compression. Figure 4 shows JPEG images with and without color reduction. Red block noise was reduced using color reduction. However, color reduction affects the diversity of colors, resulting in the loss of detail colors, as shown in Figure 4b. Therefore, we propose estimating the residual colors between images with and without color reduction to complement the color diversity appropriately.

### 3.2. Discussion of Pseudo-Contour

**Figure 5:**
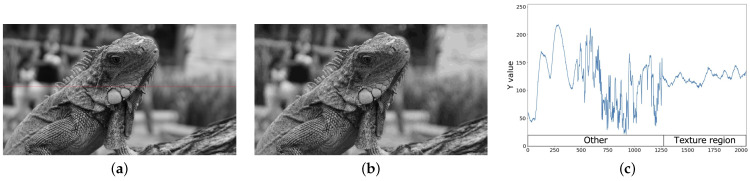
Y channels of an image containing a textured region. (**a**) Original image; (**b**) JPEG image; (**c**) Luminance values on red line.

**Figure 6:**
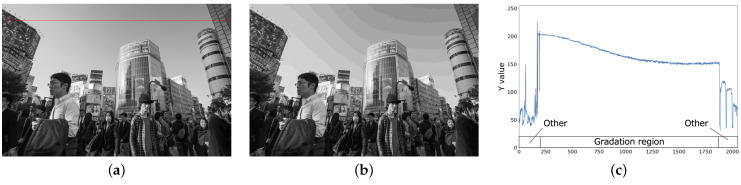
Y channels of an image containing gradient region. (**a**) Original image; (**b**) JPEG image; (**c**) Luminance values on red line.

In JPEG-compressed images, pseudo-contours mainly occur in the Y channel of YCbCr color space. Especially in the low-frequency region where luminance varies smoothly, pseudo-contouring is significantly noticeable since the quantization of JPEG coding restricts the gradation, resulting in locally discontinuous steps. In this work, we further divide the low-frequency region into texture and gradient regions. The texture region is low-frequency, and luminance changes greatly. In contrast, luminance changes smoothly in a low-frequency region. Figure 5 and Figure 6 show examples of texture and gradient regions, respectively. In the texture region, no noticeable pseudo-contour is observed even after JPEG compression while, in the gradient region, unnatural pseudo-contours are observed in the landscape image. Therefore, in order to suppress the pseudo-contour, we propose a signal-processing model that homogenizes only the gradient region of the Y channel.

## 4. Materials and Methods

**Figure 7:**
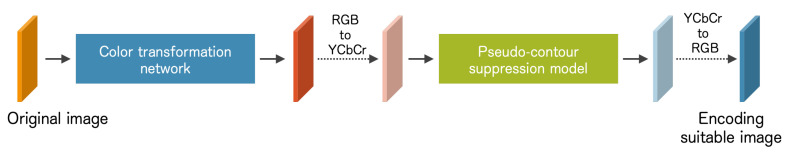
An overview of the proposed method.

The proposed method consists of a color transformation network and a pseudo-contour suppression model, focusing on unnatural tints and pseudo-contours, which are the main causes of the degradation of JPEG-compressed images. Figure 7 shows an overview of the proposed method. The input image is processed in the RGB color space for the color transformation network and in the YCbCr color space for the pseudo-contour suppression model.

### 4.1. Color Transformation Network

As we discussed in Section 3.1, color reduction from the original image can significantly suppress block noise in the red component, which is sensitive to human vision. Therefore, in this paper, we propose a neural network that extracts representative colors from an image and reconstructs the image using the representative colors.

**Figure 8:**
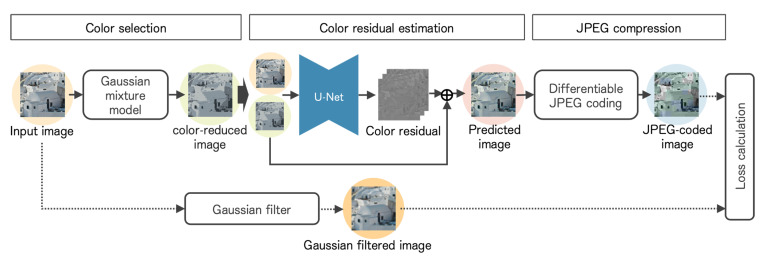
Details of the color transformation network.

The details of the color transformation network are shown in Figure 8. Firstly, we extract *N* representative colors using a Gaussian mixture model. Then, we generate a color-reduced image consisting of the *N* representative colors. Subsequently, we estimate the color residuals of the original and color-reduced images using a neural network. The proposed method adopts the U-Net architecture [22], shown in Table 1. Finally, we add the color-reduced image and color residuals to generate an image pre-processed for suppressing red block noise.

We train the color transformation network by calculating a loss value using the original image with a Gaussian filter applied (filter size 3×3). The bit rate increases when we calculate the loss using the original image. The network learns to compensate for the extra high-frequency components. Therefore, we use a Gaussian-filtered image to reduce the bit rate.

The loss functions are $L_{1}$ loss, a multi-scale structural similarity loss (MS_SSIM) [23], and perceptual loss using a VGG trained on ImageNet (VGG loss) [24]. The total loss is defined using Equation (1). We used $L_{1}$ loss since it can be expected to train the model to generate images close to the original images. Secondly, we were inspired by the work [25] of Zhao et al., and artifacts in low-frequency regions were alleviated using the $L_{1}$ and MS_SSIM loss functions. Thus, we also used MS_SSIM in this work. Thirdly, we experimentally found that VGG loss improved visual quality.

(1) $$\begin{array}{c} L_{\mathrm{TOTAL}}=L_{1}+L_{\mathrm{SSIM}}+L_{\mathrm{VGG}} \end{array}$$

We use Adam [26] as the learning optimizer. The number of learning epochs is 100. The initial learning rate is 1 × 10${}^{-4}$. The learning rate decreases to 1/10 for every 20 epochs. We use differentiable JPEG coding [27] during training. We heuristically determined the parameters in this work. Specifically, the size of a Gaussian filter, initial learning rate, and epochs are selected according to values of training loss. The thresholds of the Canny filter were determined by observing the effects of the Pseudo-contour Suppression Model on the training samples.

### 4.2. Pseudo-Contour Suppression Model

**Figure 9:**
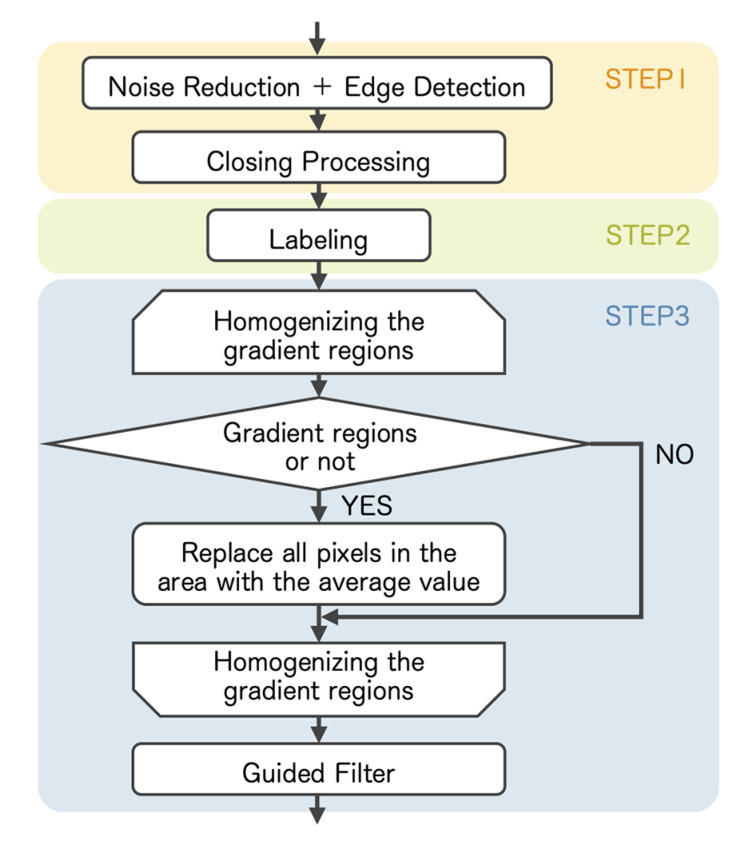
Processing flow of pseudo-contour suppression model.

The details of the pseudo-contour suppression model of the proposed method are shown in Figure 9. The input image is converted from RGB color space to YCbCr color space. We process only the Y channel of the image.

We obtain low-frequency regions using the first and second steps described in Figure 9. We use a median filter to remove noise. Then, we detect edges using the Canny method [28]. The two threshold values are set to $Th_{\min}=20$ and $Th_{\max}=30$, respectively. Subsequently, we extract high-frequency regions by applying the closing operation to the extracted edges. Finally, the low-frequency region is the bit inversion of the high-frequency region.

The third step determines the gradient regions among the low-frequency regions to homogenize it. We detect edges from the low-frequency regions using a median filter and the Canny method with the threshold values $Th_{\min}=0$ and $Th_{\max}=10$. We set the thresholds more sensitive to detect changes in the brightness of textured regions. Then, we determine the regions as the gradient regions if the number of pixels at the edges is less than 3% of the total pixels in a low-frequency region. In addition, since pseudo-contours are especially noticeable in wide gradient regions in landscape images, we added the condition that the number of pixels in each region must be more than 10% of the total number of pixels in the image to be processed. We homogenize the gradient regions using the averages. However, the homogenized regions become unnatural in the boundaries of the regions. Therefore, we apply a guided filter [29] to the homogenized gradient regions to reduce the unnaturalness of the boundaries.

## 5. Results

We conducted experiments to verify the effectiveness of the proposed method. We compiled a dataset by collecting high-quality images from the publicly available website, Unsplash (https://unsplash.com/, accessed on 1 February 2023). The training images are created by cropping 128-pixel square images from the CLIC2021. The test images are high-quality images collected from Unsplash. We selected 50 images of natural scenes, indoor scenes, animals, and buildings, since they caused red block noise and pseudo-contour through JPEG compression. Note that the training and test images are independent. Differentiable JPEG coding [27] was used only for training, and OpenCV JPEG encoder and decoder were used for testing. We trained Gaussian mixture models to extract N={4,8,16} representative colors.

The machine used in the experiments has a Xeon E5-2620, 64 GB memory, and a GTX 1080 graphics processing unit. We used Python 3.6 and PyTorch 1.10 to implement the software of the proposed method. We used the machine for pre-processing, compression, and decoding. However, we stress that powerful resources are necessary to apply the pre-processing methods to images. Decoding the pre-processed JPEG images can be performed on weak resources.

### 5.1. Numerical Evaluation

We compressed the test images using the proposed method and the standard JPEG. We set the JPEG quality to 10. The evaluation metrics were PSNR, SSIM, BRISQUE [30], LPIPS [31], and LIQE [32], which are considered to have a relatively high correlation with human perception. BRISQUE uses basic statistics, such as mean and variance of luminance values. LPIPS uses features extracted using image classification models, such as AlexNet. LIQE estimates the human perception of image quality without any reference information. BRISQUE and LPIPS are better if their values are small. Bigger values are better LIQE.

We carried out the parameter search with three values of *N* representative colors used in the color transformation. The numerical results are shown in Table 2. The standard JPEG obtained better PSNR and SSIM. Note that PSNR and SSIM do not represent perceptional quality since the algorithm is intentionally changing the input images. Thus, PSNR and SSIM do not represent perceptual performance. In contrast, the proposed method outperformed JPEG in BRISQUE and LPIPS when N= 4, 8, and 16. Thus, the proposed method focused on enhancing the visual quality related to human perception. Also, different values of *N* obtained the best results at each metric in Table 2. Specifically, N=4 achieved the best values at BRISQUE and LPIPS. The best LIQE was obtained at N=16. The difference between N=4 and N=16 is 0.016, which is a slight value. Thus, the proposed method is effective when N=4.

The results show that the LPIPS value of the proposed method is almost equivalent to JPEG when N=16. Thus, further evaluations are unnecessary, such as N=32. In this paper, we focus on pre-processing. Thus, post-filtering methods are out of scope. The comparison with JPEG is the most important for evaluation. Furthermore, we added the comparison with a pre-processing method, LearnedJPEG [11]. The details are described in Section 5.7.

**Figure 10:**
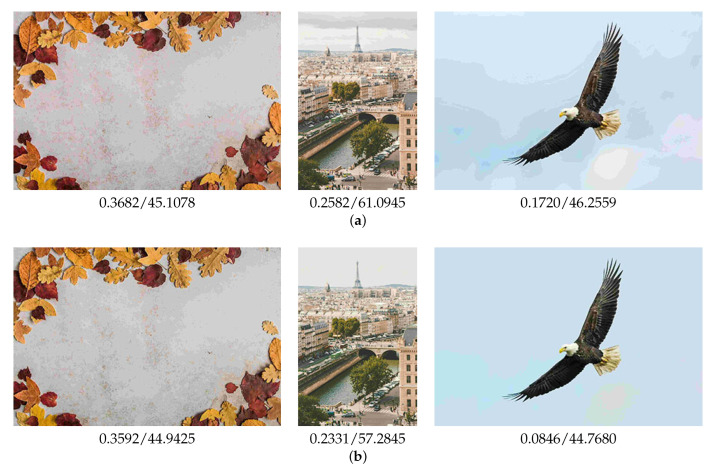
Examples of the compressed test image with LPIPS and BRISQUE values. (**a**) The standard JPEG; (**b**) The proposed method.

As shown in Figure 10, the standard JPEG coding generated block noise and pseudo-contour. Specifically, the left result had red block noise and separated colors in the background. There were pseudo-contours in the center and the right results. On the other hand, the proposed method successfully suppressed block noise and pseudo-contour, resulting in more natural-looking images.

**Figure 11:**
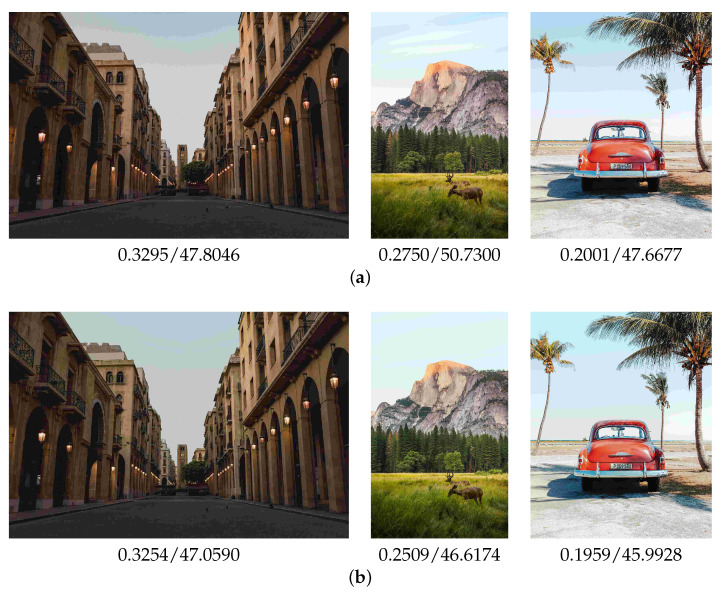
Results of sky image with LPIPS and BRISQUE values. (**a**) The standard JPEG; (**b**) The proposed method.

An artifact occurred in the right image of Figure 10b. The artifact was a pseudo-contour caused by our pseudo-contour suppression model. Comparing the right image in Figure 10a, the artifact was suppressed by the proposed method. Even though artifacts remained, the proposed method enhanced the visual quality of the standard JPEG images. The artifacts may depend on the content of a specific image. The pseudo-contour suppression model is extremely effective for the image content of the sky. Thus, we show the results of the sky contents in Figure 11. The pseudo-contours are suppressed using the proposed method. Furthermore, the LPIPS and BRISQUE values are improved.

### 5.2. Subjective Evaluation

We conducted a subjective evaluation to measure the quality of the compressed test images. Eleven participants evaluated images compressed using the standard JPEG and the proposed method with N=4. More precisely, a participant selected one answer from three questionnaires: (1) the standard JPEG is better, (2) the proposed method is better, and (3) similar quality. We determined a final answer for each test image by majority voting. For example, a test image will be answer (1) if six participants select answer (1).

Furthermore, we evaluated the final answers using statistical hypothesis testing. We set up a null hypothesis that the proportions of the three answers would be the same, i.e., one-third. Then, we rejected the null hypothesis at the significance level of 5%.

**Figure 12:**
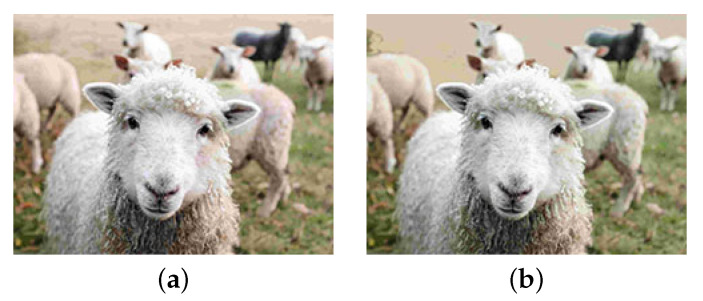
A JPEG better example in the subjective evaluation. (**a**) JPEG; (**b**) Proposed.

Table 3 shows the results. The proposed method was superior to the standard JPEG in human visual perception. The statistical hypothesis testing also verified the effectiveness of the proposed method. As shown in Figure 10, the proposed method successfully suppressed red block noise and pseudo-contour. However, there were cases where the standard JPEG was better. We showed one example in Figure 12. Although there was red block noise in the standard JPEG image, the proposed method overly homogenized the background.

### 5.3. Effect of the Color Transformation Network

**Figure 13:**
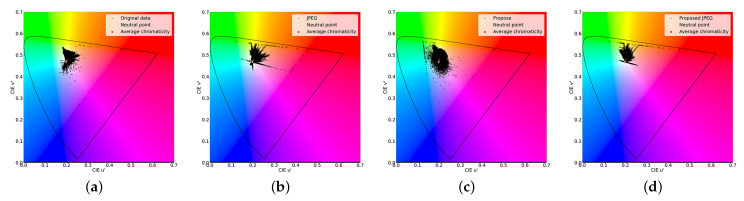
Chromaticity diagrams before and after being transformed by the color transformation. (**a**) Original image; (**b**) JPEG image; (**c**) Transformed original; (**d**) Transformed JPEG.

We applied the color transformation network to the original and JPEG images to analyze its effect. We show $u^{\prime }v^{\prime }$ uniform chromaticity diagrams in Figure 13. Comparing the original and JPEG images, red component data increased in the JPEG image and became red block noise. On the other hand, after we applied the color transformation network, the distribution of colors was shifted toward the negative $u^{\prime }$ axis by the neural network. Thus, we reduced the red component data and red block noise.

**Figure 14:**
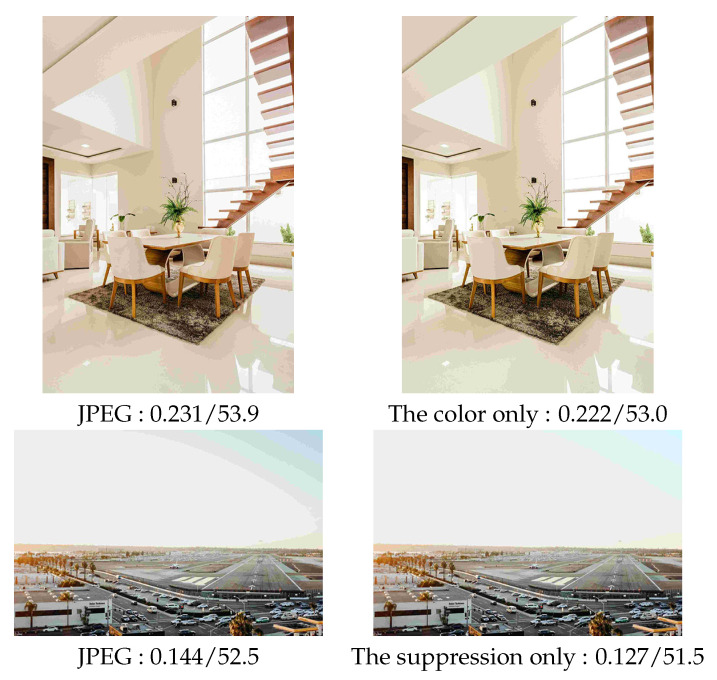
Effectiveness of the color transformation and the pseudo-contour suppression model. The values are LPIPS and BRISQUE.

We measured the LPIPS and BRISQUE to show the effectiveness of the color transformation. As shown in Figure 14, there is red block noise in the JPEG image. In comparison, the color transformation alleviated the noise. Furthermore, better LPIPS and BRISQUE values were obtained. Therefore, the effectiveness of the color transformation was verified.

### 5.4. Effect of the Pseudo-Contour Suppression Model

**Figure 15:**
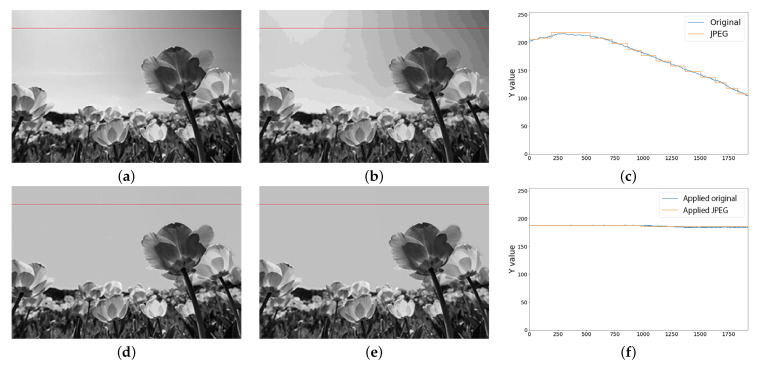
Luminance values on the red line before and after applying the pseudo-contour suppression model. (**a**) Original image; (**b**) JPEG image; (**c**) Values on the red line; (**d**) Applied original; (**e**) Applied JPEG; (**f**) Values on the red line.

We applied the pseudo-contour suppression model to the original and JPEG images. Figure 15 shows the luminance values in the gradient regions. The JPEG images had discontinuous steps caused by the quantization in the compression, and the steps became pseudo-contours. On the other hand, the proposed method homogenized the discontinuous steps, and pseudo-contours were eliminated. As shown in Figure 13, the pseudo-contour suppression model obtained better LPIPS and BRISQUE than JPEG. Therefore, the effectiveness of the pseudo-contour suppression model was evaluated.

### 5.5. Impacts of JPEG Compression Quality

**Figure 16:**
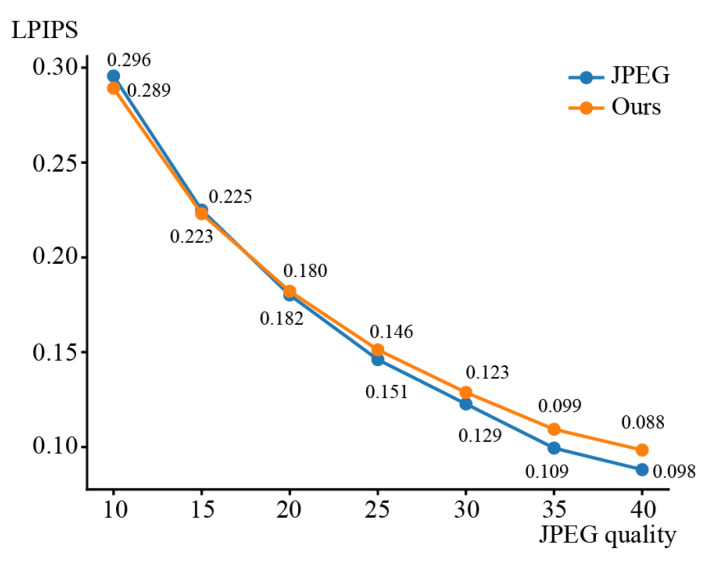
Average LPIPS at JPEG quality values.

**Figure 17:**
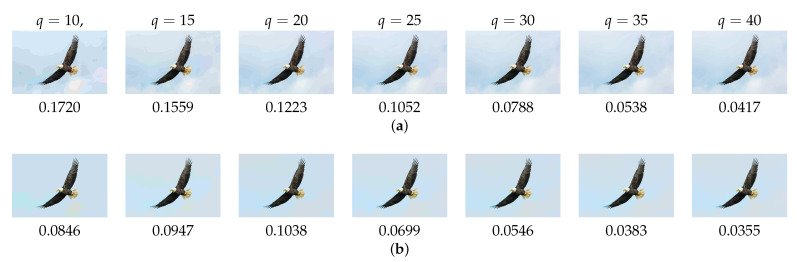
LPIPS values at various JPEG qualities *q*. (**a**) JPEG; (**b**) Proposed.

We conducted additional experiments using JPEG qualities 10, 15, 20, 25, 30, 35, and 40. We used LPIPS to evaluate the impacts of the JPEG qualities on the visual quality of compressed images. The results are shown in Figure 16. The proposed method was superior to JPEG at a quality value of 10. However, LPIPS deteriorated more than the standard JPEG from the quality of 20 and above, since red block noise and pseudo-contour occurred occasionally. We show the LPIPS values at various JPEG qualities in Figure 17. The results show that the proposed method was significantly effective at a quality of 10. Also, the values of LPIPS were improved using the proposed method. Also, LPIPS at the other qualities were improved using the proposed method.

### 5.6. Evaluation Using KODAK Dataset

The degradation of red block noise and pseudo-contour often happens in images of natural scenes, buildings, animals, etc. Thus, we developed our dataset for evaluating the performance of the proposed method, especially in the degraded images.

We conducted experiments using the KODAK (https://r0k.us/graphics/kodak/, accessed on 1 September 2023) dataset, which is used in other pre-processing methods [10,11,12]. The results are shown in Table 4. JPEG obtained better PSNR and SSIM than the proposed method. In comparison, the proposed method outperformed in terms of the BRISQUE, LPIPS, and LIQE, which are metrics for human visual perception. Therefore, the effectiveness of the proposed method is demonstrated.

### 5.7. Comparison with the Other Pre-Processing Method

We compared the results of the proposed method with another pre-processing method. We used LearnedJPEG [11] for comparison since this was the only project publicly making the source codes available. Specifically, we trained LearnedJPEG using our training data. The test images were compressed using the LearnedJPEG. The bitrates of the compressed images are larger than the proposed method. Thus, we compressed the results of the LearnedJPEG, using the standard JPEG at quality 10, for fair comparison. Note that the quality value cannot be used in LearnedJPEG.

**Figure 18:**
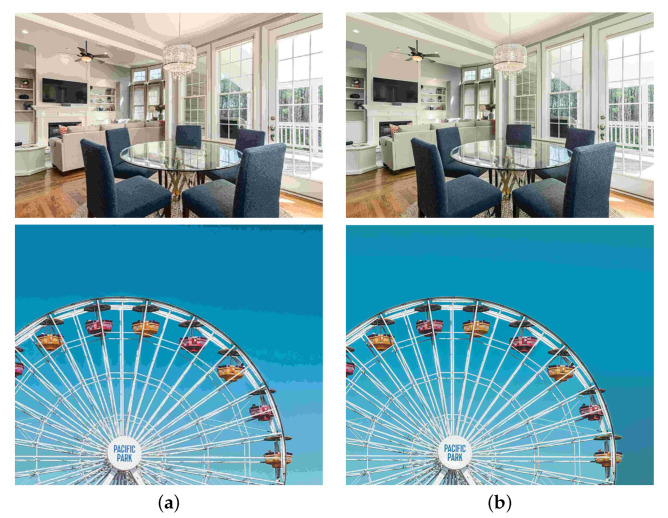
Visual comparison with the pre-processing method, LearnedJPEG [11]. (**a**) LearnedJPEG [11]; (**b**) Proposed.

The comparison results are shown in Table 5. The proposed method outperformed LearnedJPEG in all the criteria. Moreover, we compared visual quality in Figure 18. There are red block noise and pseudo-contours in LearnedJPEG. The proposed method successfully suppressed the artifacts. Thus, the visual quality in the compressed images was improved using the proposed method. Therefore, the results show the effectiveness of the proposed method.

## 6. Conclusions

In this paper, we proposed a method for generating higher-quality JPEG images without changing the JPEG decoding algorithm. We especially tackled suppressing unnatural colors and pseudo-contours, which deteriorate the visual quality of JPEG images. We proposed pre-processing to reduce colors using a color transformation network. Furthermore, we developed a pseudo-contour suppression model using signal processing. We obtained enhanced JPEG images from input images pre-processed using the proposed method. The experimental results showed that the proposed method generated images favorable to human visual perception. The subjective results showed the superiority of the proposed method. Also, the numerical results verified the competitive quality of the proposed method. In particular, the proposed method is effective for images including natural landscapes, indoors, and buildings, which are considered to be highly degraded in standard JPEG.

An essential future work is comprehensive research into the training of the proposed method using various loss functions. There are various loss functions for image quality assessment. The performance of the proposed method can be improved using other loss functions.

## Figures and Tables

**Table 1:** The architecture of U-Net used in this paper.

Components	Input Size	Output Size	Output Name
Conv2d(3,1), ReLU, BN	H×W×6	(H/2)×(W/2)×32	Conv-1
Conv2d(3,1), ReLU, BN	(H/2)×(W/2)×32	(H/4)×(W/4)×64	Conv-2
Conv2d(3,1), ReLU, BN	(H/4)×(W/4)×64	(H/8)×(W/8)×128	-
Deconv2d(3,1), ReLU, BN	(H/8)×(W/8)×128	(H/4)×(W/4)×64	Deconv-1
Concatnate(Deconv-1, Conv-2)	-	(H/4)×(W/4)×128	-
Deconv2d(3,1), ReLU, BN	(H/4)×(W/4)×128	(H/2)×(W/2)×32	Deconv-2
Concatnate(Deconv-2, Conv-1)	-	(H/2)×(W/2)×64	-
Deconv2d(3,1), ReLU, BN	(H/2)×(W/2)×64	H×W×32	Deconv-3
Concatnate(Deconv-3, Input image)	-	H×W×35	-
Conv2d(3,1), ReLU, BN	H×W×35	H×W×6	-
Conv2d(3,1), tanh	H×W×6	H×W×3	-

**Table 2:** Results of the numerical evaluation. *N* is the number of representative colors used in the color transformation. ↑ represents better value if it is larger, and vice versa for ↓. The bold and underline are the best and the second best, respectively.

	PSNR(↑)	SSIM(↑)	BRISQUE(↓)	LPIPS(↓)	LIQE(↑)
JPEG	**28.095**	**0.8745**	51.588	0.2956	1.292
Proposed (N=4)	26.924	0.8689	**51.097**	**0.2884**	1.339
Proposed (N=8)	27.239	0.8681	51.373	0.2907	**1.355**
Proposed (N=16)	26.884	0.8572	51.371	0.2959	1.345

**Table 3:** Results of subjective evaluation. The values are the number of images for each answer.

Which Is Better?	Majority Voting	Statistical Hypothesis Testing
The proposed method	30	25
The standard JPEG	13	8
Similar quality	7	4

**Table 4:** Results of KODAK dataset. ↑ represents better value if it is larger, and vice versa for ↓.

	PSNR(↑)	SSIM(↑)	BRISQUE(↓)	LPIPS(↓)	LIQE(↑)
JPEG	26.7	0.832	53.7	0.1549	1.1641
Proposed	26.4	0.828	53.6	0.1537	1.1663

**Table 5:** Comparison results with the LearnedJPEG [11]. ↑ represents better value if it is larger, and vice versa for ↓.

	PSNR(↑)	SSIM(↑)	BRISQUE(↓)	LPIPS(↓)	LIQE(↑)
LearnedJPEG [11]	19.565	0.6836	51.681	0.4505	1.022
Proposed	26.924	0.8689	51.097	0.2884	1.339

## Data Availability

The dataset used in this study will be available from the authors.
